# Witness to Harm; Holding to Account: What Is the Importance of Information for Members of the Public Who Give Evidence and May Be Witness in a Regulatory Hearing of a Health or Care Professional?

**DOI:** 10.1111/hex.14168

**Published:** 2024-08-03

**Authors:** Gemma Ryan‐Blackwell, Louise M. Wallace

**Affiliations:** ^1^ Faculty of Well‐being, Education & Language Studies The Open University Milton Keynes UK

**Keywords:** cross examination, fitness to practise, professional regulation, qualitative content analysis, witness

## Abstract

**Background:**

Health and social care regulators are organisations that seek to maintain public trust in professionals and protect the public from harmful practitioners. For example, they ensure that practitioners have the correct qualifications to practice and investigate any concerns raised about them. Serious concerns can result in a fitness to practise (FtP) hearing where a member of the public may be required to give evidence as a witness. Being a witness and being cross‐examined is known to often be traumatic, particularly for members of the public in criminal trials. There is some research evidence that registered professionals who are the subject of the proceedings may suffer mental ill health as result of the experience. But there is scant research that specifically explores the experiences of members of the public giving evidence in a FtP hearing. The regulator web pages are an important source of information for public witnesses to prepare themselves for a FtP hearing.

**Aim:**

This study aimed to examine the publicly available information for public witnesses from the 13 health and social care regulators in the United Kingdom to evaluate the content, amount, type and format of information available and make recommendations about how regulators can improve these.

**Methods:**

Regulator websites were searched during November 2021–February 2022 for information for the public on what happens after raising a concern with a regulator. Resources were downloaded and qualitative content analysis conducted. Our findings were validated by interviews (*n* = 7) with the public including people with experience of FtP and a focus group of the public (*n* = 5).

**Results:**

One hundred and forty‐six resources (97 webpages and 25 public facing documents, 20 videos and 4 easy read documents) were found. Topics included screening and investigation, preparing for a hearing, during a hearing and after a hearing, and support for witnesses.

**Discussion and Conclusion:**

We conclude that there are many deficiencies in the information content and its presentation for the public and some exemplars, such as the use of flowcharts and short videos to explain the FtP processes. Recommendations for practice take the form of a framework with three themes, (i) co‐production, (ii) preferred content and (iii) format. It may be used by regulators to enhance their support for members of the public as witnesses in FtP hearings.

**Public Contribution:**

Our advisory group of people with lived experience of involvement as members of the public in FtP discussed the findings and contributed to the recommendations.

## 1 Background

Health and social care professionals' regulators are organisations established in law to promote public confidence in the profession [1]. They hold a register of practitioners with the appropriate training and qualifications to practice their profession. Fitness to practise (FtP) refers to the health, character and competence of a professional to practise [1]. Internationally, there are over 130 regulators of nurses [2] and over 118 regulators of professions allied with medicine [3]. In the United Kingdom, there are 13 statutory health and social care regulators.

Most professional regulators have no powers of inspection but rely on people and organisations raising concerns about a health and social care professional registered with them. Concerns may include misconduct, lack of competence, criminal convictions and health conditions, amongst other matters. They have powers to remove practitioners from the register if, among other matters, a registrant causes harm or falls below standards. Concerns about practitioners may be investigated and may result in a hearing adjudicated by an independent FtP panel, where a member of the public many be required to give evidence as a witness about the registrant's conduct.

### 1.1 Literature Review

Globally, there is little research evidence that examines members of the public as witnesses in FtP hearings. Most UK research has been commissioned by regulators and includes both professionals and the public or solely professionals [4, 5, 6]. International research shows that people who have engaged with the FtP process find processes to be unfair, lack transparency and have poor methods of communication (particularly the outcome) which results in dissatisfaction, for example, not being able to understand the outcome letter [7]. This emphasises the importance of communication to create transparency about what they can expect about the hearing process.

Most research focusses on registrants' experiences of FtP procedures [5, 8, 9]. Bourne et al. [10] surveyed 7926 doctors in the United Kingdom about their experiences of complaints and concerns processes and found that they were at high risk of anxiety, depression and suicidal thoughts compounded by lack of transparency of the process particularly where the concern was investigated by the regulator [10]. Interviews with 15 nonmedical practitioners who had experience of FtP found that lack of information, length of time the process takes and poor support are the main factors impacting on feelings of anxiety, ability to cope, powerlessness and vulnerability [5].

In 2011, UK's health professions meta‐regulator (now the Professional Standards Authority, PSA), the Council for Healthcare Regulatory Excellence, published commissioned research on the experience of 25 people who had some experience of FtP, finding a lack of information and support about the process and its timelines and outcomes [11]. Research by the GMC with 44 members of the public involved in a concern with the regulator (of whom 5 had been witness in a hearing) found numerous concerns about the poor communications, disappointment with decisions and resulting distrust in the process [12]. International research with members of the public with experiences of public hearings found that people wanted to be better prepared for the FtP process [7].

The public are a substantial proportion of the sources of concern to UK regulators [13]. But current research does not focus on the information needs of public witnesses in the FtP process [4, 7, 10, 11, 14]. Being cross examined in a courtroom and the need for good quality information to prepare witnesses in criminal contexts is well established [15]. NHS Scotland interviewed victims and witnesses in the criminal justice system and concluded as part of a trauma‐informed approach and that victims of harm should be kept well informed and supported to prepare them for giving evidence based on their individual needs and circumstance [16]. Trauma‐informed practice is an approach which is grounded in the understanding that exposure to trauma can impact on an individual's biological, social and psychological development [17]. The provision of accurate and complete information is therefore a fundamental component in preparing and supporting people who have been harmed to be witnesses [16, 18]. Transparent, well designed, accurate and trustworthy information has also been shown to be an essential requirement for building and maintaining public trust in organisations (a primary purpose of regulators) [19, 20, 21, 22].

This study aimed to examine the publicly available information for witnesses from the United Kingdom's 13 health and social care statutory professional regulators to evaluate the amount, type and format of information available and make recommendations about how regulators could improve the information available to support and prepare people after raising a concern with a regulator.

## 2 Methods

### 2.1 Design

A qualitative content analysis was applied to downloadable documents, webpages and videos on the 13 UK statutory professional regulator websites.

### 2.2 Data Collection

#### 2.2.1 Content Analysis

Data collection took place between November 2021 and February 2022 (this time period was selected as this project was part of a larger programme about the public's role in fitness to practise). Webpages and downloadable documents were saved as portable download files (.pdf) and uploaded to the qualitative analysis software NVivo 12.0. Video transcripts were included with the documents to analyse the content. Regulator websites were used to search for any information (including downloadable documents, Easy Read documents and webpages) aimed at members of the public once they have raised a concern including the following topics:

a. Giving a witness statement in the investigation process.
b. Being a witness at a hearing.
c. Being cross examined.
d. Support available for witnesses.
  i. Regulator.
  ii. Independent.

Resources were excluded if they were only aimed at registrants, expert witnesses or employers.

The 13 regulators are shown in Table 1.

**Table 1:** The 13 UK regulators.

Regulator	Acronym
General Chiropractic Council [23]	GCC
General Dental Council [24]	GDC
General Medical Council [25]	GMC
General Optical Council [26]	GOC
General Osteopathic Council [27]	GOsC
General Pharmaceutical Council [28]	GPhC
Health and Care Professionals Council [29]	HCPC
Northern Ireland Social Care Council [30]	NISCC
Nursing and Midwifery Council [31]	NMC
Pharmaceutical Society of Northern Ireland [32]	PSNI
Social Care Wales [33]	SCW
Scottish Social Services Council [34]	SSSC
Social Work England [35]	SWE

The tribunal services of GMC and HCPC regulators were also included (at the time of the analysis, the Dental Professionals Hearing Service website was not live):

- Medical Practitioners Tribunal Service (MPTS).
- Health and Care Professionals Tribunal Service (HCPTS).

These are organisations established to maintain independence between the regulator and the investigation of concerns.

#### 2.2.2 Focus Group and Interviews

An anonymous focus group and interviews were conducted to explore the usefulness of information offered to the public including those who have previously raised a concern with a regulator. Recruitment was through patient participation groups of the GPhC and SWE and a UK charity, Action against Medical Accidents (AvMA). These were recorded and transcribed.

Participants recruited from the regulators were asked to do a preparatory exercise which involved looking at several regulator documents and videos and answering questions about them which would prompt discussion in the interview or focus group about the materials they had being provided with. They were not asked to undertake research by looking at the regulator's website directly, as it was important that all participants who had no prior experience of FtP would be commenting on the same stimulus materials. This approach was not used for participants recruited from the third sector organisation, as it was anticipated they would have direct personal experience which would be more relevant for them to discuss.

### 2.3 Method of Analysis

Qualitative content analysis as described in Altheide and Schneider [36] was applied as it was specifically designed for qualitative analysis and derivation of meaning of different types of media/information. This is a 12‐step process that develops, tests and applies a coding framework. Stage five ‘tests’ the coding framework, and at this point, it was identified that further data about the format of web pages were required. The focus group and interview transcripts were tabulated under the themes such as information needs [content], format and support at each stage after raising a concern.

### 2.4 Ethical Considerations

The documents and webpages accessed were all available to and intended for use by the public via regulator websites and the regulators.

Ethical approval was granted by the host university Human Research Ethics Committee (HREC/4058). Participants in the focus group were anonymised so they were not individually identifiable; interviewees' identities and case information were anonymised.

### 2.5 Reliability of Coding and Validity of Initial Findings

Three members of the research team reviewed the included documents found via the NMC website and agreed a coding framework to be applied to the other included documents. The NMC was chosen as the largest regulator in the United Kingdom and the one with the largest number of documents.

Preliminary findings were presented to an online focus group with members of the public with experience of healthcare complaints (*n* = 5). Each group member was given a summary of the findings and completed three activities that asked them to make notes about their opinions of these findings. These were then discussed during the focus group to allow the team to validate the findings about what content, volume and format is viewed to be effective in promoting public trust in and understanding the FtP process and the role of a public witness. Interviews were also conducted with seven members of the public involved as members of regulators' public forums. These were recruited via AvMA (interviewees 2–5), two from SWE (interviewees 1 and 6) and one from GPhC (interviewee 7).

## 3 Results

In total, 146 resources consisting of webpages, leaflets and video content were included in the analysis (see Table 2).

**Table 2:** A summary of included resources by type and regulator.

Regulator	Number of downloadable leaflets (e.g., pdfs/word docs)	Number of documents created from webpages	No. of videos	Number of easy read documents	Total resources
GCC	2	3	0	0	5
GDC	2	3	3	0	8
GMC	0	15	2	0	17
GOC	2	6	0	0	8
GOsC	1	4	1	0	6
GPhC	5	5	0	0	10
HCPC	1	6	0	0	7
NISCC	2	2	0	0	4
NMC	4	18	11	4	37
PSNI	1	0	0	0	1
SCW	1	1	1	0	3
SSSC	1	4	1	0	6
SWE	0	9	0	0	9
MPTS	2	9	1	0	12
HCPTS	1	12	0	0	13
Total	25	97	20	4	146

Table 2 shows the volume of public focussed materials ranged from a single leaflet of the PSNI to 15 documents and 2 videos by the GMC.

Table 3 provides a summary of the content found for each regulator and tribunal service. The associated regulators provided some information about each these stages. As expected, the MPTS and HCPTS did not provide information about screening and investigation.

**Table 3:** A summary of content found across all formats by regulator.

Stage	Regulator	GCC	GDC	GMC	GOC	GOsC	GPhC	HCPC	NISCC	NMC	PSNI	SCW	SSSC	SWE	MPTS	HCPTS	Total
Screening ad investigation	Giving a witness statement	✓		✓	✓	✓	✓	✓	✓	✓		✓	✓	✓			11
	Regulator support/point of contact (e.g., case examiner)	✓	✓	✓	✓	✓	✓	✓	✓	✓	✓	✓	✓	✓			13
	Independent support		✓	✓	✓		✓	✓		✓				✓			6
Before a hearing	Practical advice (e.g., expenses, travel)	✓	✓	✓	✓	✓	✓	✓	✓	✓		✓	✓	✓	✓	✓	14
	People involved	✓	✓	✓	✓	✓			✓	✓	✓	✓	✓	✓		✓	14
	Regulator support	✓	✓	✓	✓	✓	✓	✓	✓	✓		✓		✓		✓	12
	Independent support	✓	✓	✓	✓	✓	✓	✓		✓					✓	✓	91
	Preparing for a hearing (e.g., Do I have to attend? When and where?)	✓	✓	✓	✓	✓	✓		✓	✓	✓		✓	✓	✓	✓	13
During the hearing	Regulator support		✓	✓	✓	✓			✓	✓			✓		✓	✓	9
	Giving evidence and cross examination	✓	✓	✓	✓	✓	✓		✓	✓			✓	✓	✓	✓	12
	Who is involved?		✓	✓	✓		✓			✓	✓	✓	✓	✓	✓	✓	11
	Hearing room layout	✓	✓	✓	✓	✓	✓	✓		✓							8
	Virtual/interactive tour				✓	✓				✓						✓	4
After the hearing	Giving feedback	✓	✓	✓	✓	✓	✓	✓	✓	✓			✓	✓	✓		11
	Possible outcomes	✓	✓	✓	✓	✓	✓	✓	✓	✓			✓	✓	✓	✓	12
	Regulator support		✓	✓		✓			✓	✓				✓	✓	✓	8
	Independent support		✓					✓							✓	✓	4

### 3.1 Screening and Investigation

#### 3.1.1 Information About Process

All regulators provided some information about the screening or investigation stages of the process. The process itself varied between regulators; so, this information reflected this variation. For example, GPhC [37] *Guide to giving a witness statement* covered each of the four stages they follow and what happens in each, along with possible outcomes following the investigation and what these mean for the registrant. This document used a flowchart and supporting narrative to outline the process and explicitly stated how long it would typically take to complete the process. Focus group participants and interviewees valued transparency about the length of time the process would take and the use of flowcharts to summarise a complex process

I think you need also to have a good understanding of the process and what you may be getting yourself into and the before, during and after scenarios…How long, what would be the format, how long are we talking about?

(Focus Group, FG)

They'd got flowcharts about the process. It was very good, I thought the [GMC] website was quite good, to be honest.

(Interview, I, 4)

By contrast, the GCC [38] provided a webpage that summarised the process in far less detail, with a bullet pointed list and language referring to the ‘decision’ stage of an investigation without explanation about what could be decided and when. This lack of specific content including timescales for decisions in regulator information caused frustration especially for those with prior experience of the FtP process.

It was frustrating in the timescales they gave and also when we spoke to them about each step of the way it was very frustrating hearing that.

(I4)

#### 3.1.2 Information About the Witness Statement

Eleven regulators mentioned that there may be a need for those who raise a concern to provide a witness statement as part of the investigation process. This varied between regulators, with some giving it a simple ‘mention’ without being explicit about what it would entail, what the purpose of the statement was, how it would be used and the importance of telling the truth.

Others were more transparent about the importance of the witness statement. For example, the NMC [39] *Being a witness: Your part in an investigation* which is a 13‐page document explains specifically about this stage of the process. Importantly, this NMC document along with five other regulators provides information about independent organisations that can offer support. However, this NMC document was not specifically written for members of the public as witnesses but for any witness required to provide a statement as part of the investigation stage. Transparency was also viewed by interviewees to be of significance in building trust in organisations and services [19, 20, 21],

Even trust is important for me and even if you can feel that you trust them like I felt that I could trust GMC.

(I7)

### 3.2 Preparing for and During a Hearing

All regulators provided some information to prepare for a hearing. This most often included practical information such as,

– Reimbursement for expenses.
– Attending a virtual hearing.
– When and where will it take place.
– Who will be present?
– Do I have to attend?

Focus group participants stated that this is the type of information they would need, along with support during the hearing,

How long? What, what? What would be the format? You know, how long are we talking about? How long is this gonna take to you know what I mean? How much? How much time, you know, if you're not. Well, does he have to help? Could you attend meetings in Zoom? Are you expected to turn up in person?

(FG)

Twelve regulators provided practical information, for example, how to claim expenses and six had a video or virtual tour of the hearing room, with five having resources showing the layout of the hearing room. Of the virtual versions (*n* = 3), the tours were interactive so that the user could click onto areas and people and walk around rooms, such as that from HCPTS [40] *Virtual tour.* Similar approaches have been used in education for many years, and research suggests that this type of resource has benefits for people with reading and writing difficulties, specialist learning needs or language barriers and their positive role in promoting retention of information [41, 42]. Focus group and interviewees felt that the virtual tour would reduce their anxiety about attending the hearing,

they have a 3D room which gives you the, well it gives you the example of a hearing and what that looks like in that room. And actually, it very much reflected my experience …. It gave you indicators to who was going to be sitting where and what their purpose and role was, which was very reassuring for me, because I was quite nervous about numbers of people who would be present.

(I6)

Given the shift to remote hearings because of COVID‐19 restrictions, six regulators/tribunal services contained some information about attending virtual hearings, for example, how to use Microsoft Teams. However, some of these resources were very detailed (MPTS) [43] and could be overwhelming to people who do not have a high level of digital literacy. Focus group and interviewees noted that resources and information need to be inclusive,

you know people will have different level of of reading and language skills. So actually being able to have the information kind of written in different formats is useful.

(I1)

Ten regulators provided information about who would be in the hearing and what their roles and responsibilities were. Focus group participants felt that this was important but also would like to know the names of people who would be present before the hearing, for various reasons, including the need to know it would be a ‘fair hearing’ without any conflict of interest and that the hearing would proceed and not be abandoned. And this is fundamentally important in promoting trust [19, 44],

To make sure there's no conflict of interest and that you're well prepared for who that person is going to be, who's those people are going to be.

(FG)

However, there were deficiencies in the content about enabling witnesses to understand the conduct of a panel and what is expected of them.

Focus group participants and interviewees suggested that a video (or series of short videos) simulating an actual hearing would help to prepare them,

And at practice, video that showed the whole procedure of the thing. That's not actually a real case, but the whole procedure of the thing, you can visualize because that's a big thing.

(FG)

Given that our interviewees found giving evidence to be a challenging process, the use of a video would potentially illustrate the reality of being cross‐examined,

it's about everything you've said and having it pulled apart. Every single word of every sentence questions challenged taken apart by a barrister who is looking to find holes in your story … But I think it needs to be exactly how it is and might be

(I7)

How they had worded the question was quite convoluted to be honest and I just remember it was a really particularly long question that I had to ask him to rephrase it because I really didn't understand what he was trying to ask me. And also, I don't know, this might have just been me, but I'd forgotten that I wasn't allowed to refer to my notes. So I'd gone to have a look and they were like no you can't re‐read them now, that was it…I can actually visualise it in my head now being sat in that chair and the panel members in front of me, in front of the glass, and the nurse was literally just sat about two or three seats away from me. So yeah definitely, it was a hot seat for sure, yeah.

(I6)

All regulators provided some information about the hearing, but the level of detail varied. However, eleven regulators noted that there would be a key contact to liaise with the witness before the hearing. Whether they would have a role in practical matters or about the procedure and presentation of evidence was not explained.

**Figure 1:**
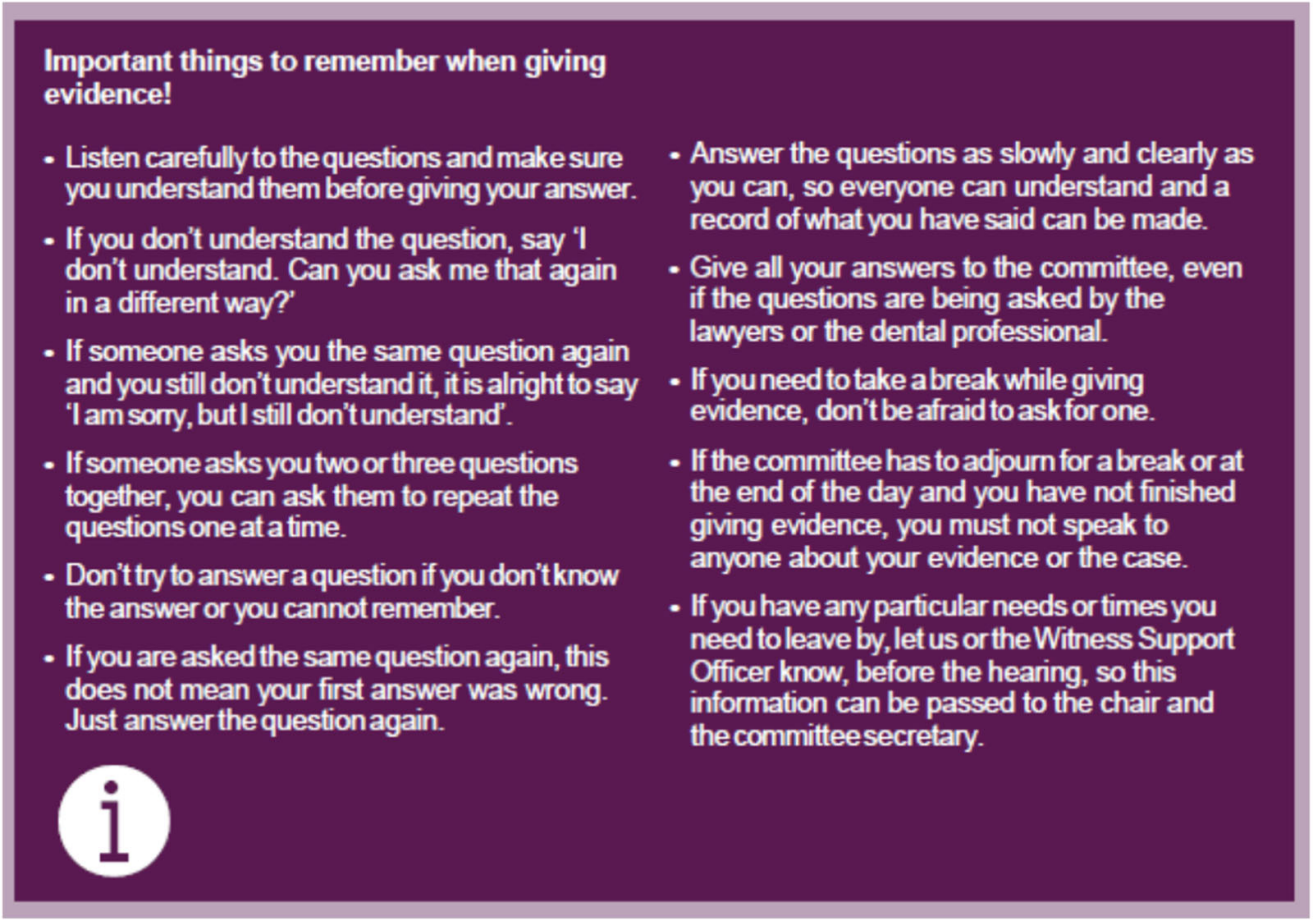
GDC key things to remember when giving evidence.

The NMC [39, 45] documents *Giving evidence and cross‐examination* (website) and *Being a witness. Taking part in a hearing* explain the process of cross‐examination and helps the reader ‘prepare’ to give their evidence. The GDC [46] explained ‘what happens in the hearing room?’ and ‘how the questions will be asked?’ which takes the reader through what will happen step‐by‐step and quick reference list/summary guides (Figure 1).

While it is acknowledged that there would be verbal explanations by regulator staff about the hearing, it is important that these are reinforced with written information [12, 15, 19].

None of these resources explained the inquisitorial nature of questioning, that is, that their evidence would be tested, but interview participants felt that this was essential information,

I would like to talk to the person who's going to be the prosecutor if you like. I'd like some idea of how it's going to come across, how am I going to be, and that's why I'm going to try and go to these tribunals before and see the process so I get some idea of whether they're trying to trick you, whether the questions, a bit like your question there is a bit of a naughty question, whether they're trying to trip me up or anything like that.

(I4)

Most but not all regulators have powers to summon a witness to give evidence, including the public. Most noted it was important for the public to give evidence, but only 4 regulators' public focussed documents describe this legal power.

### 3.3 After a Hearing

Ten regulators provided routes for people to give feedback about their experience either via a survey or the witness support in person, and 12 regulators provided information about the possible outcomes/sanctions that could be imposed and how this would be communicated to the witness, usually in writing and the possibility of a registrant appealing the panel's decision. Focus group participants welcomed this,

After a decision was made at ftp hearing I would want the full judgement result in writing whether or not the decision went in my favour.

(FG)

Information about support was only offered by 7 regulators and primarily directed people to their case worker (or equivalent) and included practical information about claiming expenses, for example GOsC [47] *Witness Guidance* document.

In summary, the information about what happens after a hearing was limited to short paragraphs of information including sentences about the following themes:

i. Claiming expenses.
ii. Outcome decisions.
iii. Registrant appeals.
iv. Signposting to a case worker or equivalent (depending on the regulator).

### 3.4 Format, Length and Target Audience

#### 3.4.1 Format

While most of the content was in text form, five regulators provided a flowchart that summarised the whole process from submitting a complaint through to hearing/post hearing. Flowcharts delivered information in a concise and logical manner without a significant amount of narrative. The use of ‘symbolic’ content such as images has been shown to promote ‘belief’ and trust in the content presented [22].

Focus group participants and interviewees reflected the need for information to be provided in different formats and that flowcharts, images [with clear purpose], videos and ‘scenarios’ as examples were particularly useful,

You need a website which you can download documents which have all the information because lots of people can't read online very easily on for some whatever reason and they like to have something in hand so they can refer to on any occasion, you know when [in] the sitting [hearing]. Some people find it helpful to look at, pictures …, videos are good idea as well because we lots of people go to YouTube for instructions …

(FG)

**Figure 2:**
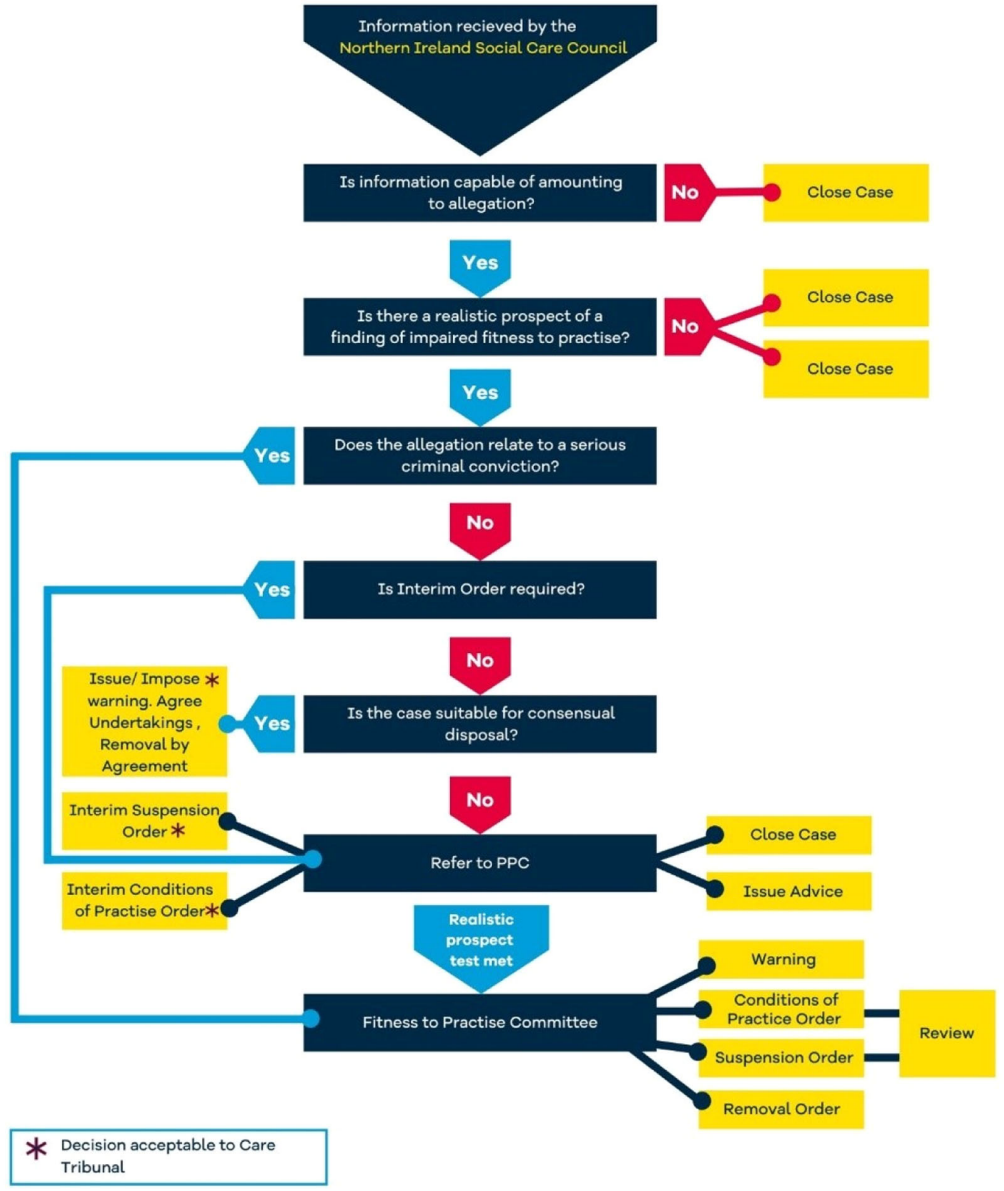
A step‐by‐step guide.

The NISCC [48] provided a ‘step‐by‐step’ guide that provides an overview of the process in a simple way, although it does indicate what will be required from the member of the public (Figure 2).

However, this image was embedded into a 20‐page document, rather than providing an initial overview. Following the suggestions of the focus groups and interviews, it could be used to summarise and then signpost more detailed text information.

Five regulators used videos and six provided virtual tours of the hearing room to summarise key points members of the public in the focus group and interviewees found useful,

They give you a virtual tour of the room and all that, yes … (helpful?) Yes I did yeah.

(I5)

#### 3.4.2 Length and Amount

SCW [49] provides a video on the FtP process but is over 17 min long. Although it is possible to pause and revisit videos, best practice for the delivery of information through videos suggests that they should be no longer than 6 min and where videos go beyond this, they should be segmented [50]. However, there were some issues with accessibility with some of the videos such as the absence of a transcript and the option for subtitles [51]. The GMC's videos met these requirements.

There were some lengthy documents that described the whole FtP process in detail, while others were more specific to giving evidence in a hearing, for example, GDC [46] *Witness information guide* (24 pages), SSSC [52] *Raising a concern: guidance for members of the public and colleagues.* The focus group feedback suggested that people would want to know what would happen after making a complaint, but some of these documents relating to being a witness contained excessive detail about the process and could be more clearly designed to be read by those who are eventually confirmed to be a witness, for example, NMC [39] *Being a witness: taking part in a hearing* (19 pages). However, some of these longer documents contained contents pages (GDC [46] and SSSC [53]), which is good practice as it helps the reader identify the information they require rather than reading the whole document and reflects UK Government guidance [54, 55].

Some regulators have large numbers of documents, webpages and videos available, such as the NMC (*n* = 37) and GMC (*n* = 17). While information was welcomed, too much information on the same topic was viewed by interviewees as overwhelming,

and if they're crammed with too much information then they put off don't they?

(I3)

#### 3.4.3 Audience

A example of clarity about the focus of information for the public was found in several documents, such as that from SCW [56] which states,

You may be raising concerns about your own care or acting on behalf of a friend or relative, or as a carer.

There were several regulators that produced documents or website information where the target audience was unclear or there was a mixed audience, with some documents that appeared to be aimed at both employers and members of the public [53].

Some documents seemed to be public facing but contained high‐level language or were more about policy rather than for the public, for example, PSNI [57] ‘Remote hearings Standard Operating Procedure’. The SSSC [58] *Are you attending a hearing?* seems to be for a range of audiences and lists items as ‘registered workers, witness for SSSC, witness for a worker’ and only then followed by ‘a member of the public wishing to attend a hearing’. It also refers to ‘media wishing to attend’.

The implications of having materials that do not explicitly inform the public as the key audience may be confusing and devaluing, impacting on the development of trust in the organisations outward facing priorities, that is, it is suggesting its priorities are employers or registrants rather than primarily the public [19].

## 4 Discussion: The Role of Information in Building Trust

Our study has shown that the public and people who have experienced the FtP process had mixed experiences of the information provided to them and had varied and individual preferences about the content, format and type of information they want available to them, particularly in preparing them for the FtP process and being a witness at a hearing.

Research referred to above shows that there is a need for more preparatory information for members of the public, who are required to become witnesses in a public professional regulatory hearing. This study is novel in that it looks specifically at the content of preparatory information for public witnesses, across multiple regulators of health and social care and tribunal services in the United Kingdom.

Regulators should consider offering the same information in multiple formats to best meet the needs of diverse members of the public including written information. It should be created for the audience in question, which could be improved by co‐production of information aimed at members of the public specifically. The text should cover the whole FtP process after a concern is raised and the support offered, with text design including the use of short summaries and bullet points. The format should include .pdf format, websites and visual and audio methods.

A new approach to the preparation for witness cross‐examination would be a mock cross‐examination animation or video role play. This could include to include the type of questions asked. It could also include indicating how the panel members and lawyers can be expected to behave. This may improve the witness experience and witness engagement. It may also reduce the feelings of vulnerability and anxiety and enable them to respond more effectively and accurately to questioning. It should contain information about what cross examination is, types of questioning and what they can do to exert control (such as asking the panel if a line of questioning is relevant) to improve agency and empower the witness to feel more in control. The video should comply with government standards for accessibility, including subtitles and a transcript [51]. Information should be accessible at the point the person chooses. For some, this may be well in advance of the hearing and would allow people to revisit the content for repeated exposure. This may improve the witness experience and engagement. It may also reduce the feelings of vulnerbility and enable them to respond more effectively and accurately to questioning.

Most regulators do not address the power imbalance inherent in the regulatory process. As described above, content for the public does not make it clear whether public witness participation is voluntary. The content also gives no attention to agency for the witness to understand the purpose of inquisitorial cross‐examination and whether they have to respond to questions, or for example, that a line of questioning is stopped or rephased. Previous research by the HCPC of the determinations (outcomes) of sexual abuse cases against social workers describes the intrusive testing of victims' truthfulness as a witness: the regulator's failure to protect witnesses from harm [59].

UK and international studies [7, 14, 60, 61] highlight that the public are the largest source of concerns raised wth regulators; yet, these cases are far less likey than those from other sources to be taken to a hearing for an outcome, which has been suggested to be in part due to not only an inherent power imbalance between professionals and the public but also large variation in familiarity with regulatory process (regulatory literacy).

Our study goes some way to suggest, at a very practical level, this power imbalance is exacerbated by inadequate and inaccessible information provided by regulators. However, the public may provide crucial factual evidence to help regulators and their FtP panels to understand what has happened when a concern is raised about a professional's behaviour. Attending to the public's (and harmed colleagues') perspectives is of relevance to the regulators' purpose which is to protect the public [62]. The engagement of the public is therefore not only relevant to regulators' overall purpose but also is crucial the protection of the public since if the public do not engage or, having engaging but withdraw before or during a hearing, this could lead to the collapse of a case, as well as to building distrust in professional regulation and in the professions and in the services they provide [59, 62].

**Figure 3:**
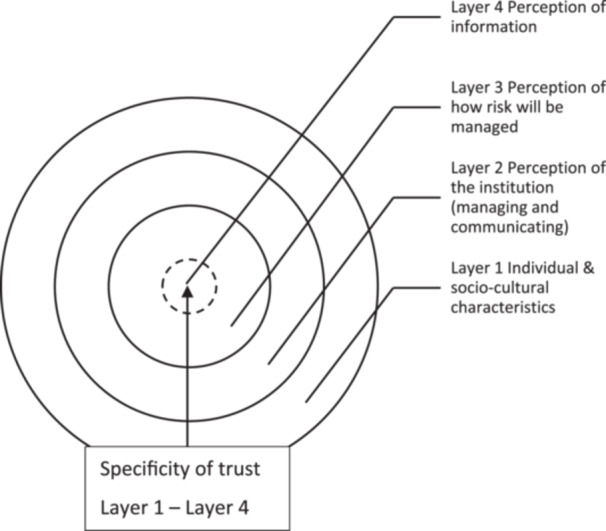
Layers of trust related factors in communication (adapted from Chryssochoidis et al. [19]).

Chryssochoidis et al. [19] integrative review and associated findings present a conceptual framework of layers of trust (Figure 3). In the context of our study, layer 1 is considered in a broad context and dependent on the characteristics of the person who raised the concern. This includes aspects such as the trauma they or their family member have experienced, their motivations for raising the concern and the broader experiences of their life, for example, educational level and social economic status. This emphasises the need for regulators to consider the individual needs of witnesses in relation to content, format and support during the FtP process and is reflected in our findings. Layer 2 involves the concept that institutions that are sources of information because of circumstance, which in the context of our study, regulators that require a person to be a witness for the regulator. From our findings, it is argued that, to promote trust, regulators need to demonstrate competence, expertise and willingness to protect the public by providing transparent, honest and fair information to people become witnesses. Layer 3 refers to risk (risk of engagement/withdrawal); in the context of our study, this refers to the FtP process (or public perspective of it) and how this is managed and communicated. It refers to the risk that poorly designed and communicated information could increase the risk of mistrust or lack of confidence in the regulator, its processes, any outcomes and witness disengagement. Layer 4 refers to the perception of the information received by the individual or public which is the subject of this study and the content to be presented in more than one format, allowing for variations in the volume of content which our study has explored in detail.

We therefore argued that key information relevant to engagement with investigations and being a witness should address all these layers to facilitate decisions of the public about witnesses' involvement. As an example, the content of regulator information was unclear as to whether the participation is voluntary or whether compulsory measures are ever put in place.

### 4.1 Recommendations

By triangulating findings from Biggar et al. [7], our study and wider research literatures [4, 12, 16, 18] make evidence‐based recommendations based on a logic model [63], which can be made about what regulators may be able to do to improve the experiences for witnesses through the provision of information. This should be part of a wider trauma‐informed approach (Table 4) under three main themes, preferred content, format and co‐production and collaboration. Co‐production and collaboration engage stakeholders at the earliest stages of design and development; it acknowledges that people with lived experience are best placed to advise on content, format and approach to the delivery of information [64]. There are a range of co‐production models available, and these should be selected based on desired outcomes and circumstances [64, 65].

**Table 4:** Recommendations: improving the provision of information to prepare witnesses for FtP hearings (adapted from Bigger et al. [7]).

	**Preferred content**	**Format**	**Co‐production and collaboration** [16, 18, 66]
What can be done to improve?	– The purpose of regulators; what they are and are not – Your choice to attend a hearing (compulsion) – What ‘special measures’ can be put in place – Practicalities of attending a hearing – Support provided by the regulator and independent services – Processes and procedures including timeframes for providing information and for receiving decisions – Communication strategies and lines of communication. What, how and when? How frequently? – Cross examination., what it is and what it isn't – What you can and others can do to manage how you are cross examined – Outcome. What are the possible outcomes, what do they mean for everyone involved?	– Various formats available to deliver similar information in different circumstances, e.g., flowcharts illustrating process, videos, images, leaflets and booklets, role‐play/interactive practice for cross examination – Video and virtual tours. – Video of a ‘typical’ hearing or cross examination. – Follow UK Government [51, 54] guidelines for websites and accessible documents but ensure that options are available for people who want to download information, for example – Consider the length of written content and videos – Provide multiple channels of communication including information in alternative languages representative of the used in the wider population demographic – Consider the potential of Artificial Intelligence platforms for the delivery of information, e.g., translation [67] functions	– Involve previous witnesses and members of the public to produce fit for purpose information – Use evidence‐based tools and processes such as Patient Information Forum [65], Thinklusive [68] and NHS [69] to guide the process and ensure that it is genuinely ‘co‐produced’ and tailored to the audience for which it is intended
Generic interventions	Examples taken from transferable interventions [16]: – Training and professional development for those who manage FtP processes and communication with members of the public. About how to make the process tailored to individual needs and the experiences of members of the public, which will in turn promote a positive culture within FtP regulator teams and promote a learning environment		
Objectives of improved information for the public	– A single pint of contact within the regulator – Ensure people always have access to independent advice and support [16, 18] – Produce information in the right format, for the right audience and the right time in the FtP process – Promote effective and transparent communication strategies to enhance trust and confidence in regulators and regulatory processes – Acknowledge that the outcome is important to witnesses who are members of the public. Provide honest and clear information about the outcome and why the decision was made – As early as possible provide information that assists witnesses about realistic expectations of what is expected of them what support they can access and what the outcomes might be		
Proposed outcomes: – Promote trust, agency and confidence for witnesses who are going to be cross examined and empower them to take on the role to their benefit which may lead to increased trust in regulation and continued engagement in FtP processes by the public, for the public good			

### 4.2 Impact and Limitations

Our research is confined to statutory regulators and tribunal services for health and social care in the United Kingdom and did not consider the voluntary accredited registers in the United Kingdom, nor did it make international comparisons. This study included information available during a fixed period of time, and soon after this, a new hearings website was launched for the dental professionals registered under the GDC. The regulators were offered a regulator specific report including comparisons across regulators and a workshop to explore the results, which was taken up by 11 or the 13 regulators. Since this time, several of the regulators have made minor or significant amendments to their websites and documents (e.g., NMC and GoSC), which illustrates the evolving nature of information available.

It is noteworthy that the UK's meta‐regulator, the Professional Standards Authority, compares non‐UK regulators' approaches and processes in FtP against the UK standards [70, 71], providing an impetus for improvement which may in future include addressing the issues raised in this study.

However, the findings of this study will inform regulatory bodies in other countries in providing information to support and prepare witnesses for FtP hearings and could apply to regulators in other service sectors, such as accountancy or veterinary medicine. This study has shown the importance of information in promoting public trust in regulators which situates itself in the emerging research in this field. No such study has explored the role of information in promoting public trust in the FtP component of professional regulation.

## 5 Conclusion

For the first time, this study has examined the content of documents and webpages intended for members of the public who have raised a concern to a regulator and in preparation for being a witness at a FtP hearing. It applies a trust theory that illustrates how information and the way it is delivered is fundamental in promoting public trust in regulators FtP processes and in preparing them to be a witness at a FtP hearing. This trust theory illustrates the important role regulators have in managing and communicating with members of the public about the FtP process. Recommendations for practice take the form of a framework with three themes, (i) coproduction, (ii) preferred content and (iii) format. It may be used by regulators to enhance their support and communication for members of the public as witnesses in FtP hearings.

## Author Contributions

**Gemma Ryan‐Blackwell:** methodology, validation, investigation, funding acquisition, writing–original draft, writing–review and editing, project administration, data curation, formal analysis. **Louise M. Wallace:** investigation, conceptualisation, funding acquisition, methodology, writing–original draft, validation, writing–review and editing, project administration, supervision.

## Ethics Statement

The Open University Human Research Ethics Committee HREC/4058.

## Consent

Participants provided valid informed consent.

## Conflicts of Interest

Louise Wallace is a Lay Panel member for the General Dental Council and Lay Adjudicator for Social Work England.

## Data Availability

Data from this research are available by contacting the corresponding author.
